# Coenzyme Q10 improves ovarian histology and attenuates the expression of angiogenesis-associated proteins in the ovary of rats with experimental hyperstimulation syndrome

**DOI:** 10.22038/IJBMS.2022.64010.14096

**Published:** 2022-08

**Authors:** Zahra Darabi, Zahra Basir, Mohammad Reza Tabandeh, Zohreh Ghotbeddin

**Affiliations:** 1 Master of Histology Student, Faculty of Veterinary Medicine, Shahid Chamran University of Ahvaz, Ahvaz, Iran; 2 Department of Basic Sciences, Faculty of Veterinary Medicine, Shahid Chamran University of Ahvaz, Ahvaz, Iran; 3 Department of Biochemistry and Molecular Biology, Faculty of Veterinary Medicine, Shahid Chamran University of Ahvaz, Ahvaz, Iran; 4 Stem Cells and Transgenic Technology Research Center, Shahid Chamran University of Ahvaz, Ahvaz, Iran; 5 Department of Physiology, Faculty of Veterinary Medicine, Shahid Chamran University of Ahvaz, Ahvaz, Iran

**Keywords:** Angiogenesis, Cabergolin, Coenzyme Q10, Gene expression, Histomorphology ovary, OHSS

## Abstract

**Objective(s):**

Ovarian hyperstimulation syndrome (OHSS) is an iatrogenic complication characterized by many side effects. Coenzyme Q10 (CoQ10) is a protective lipophilic molecule with an extensive range of biological functions, but its possible protective effect on the ovary in OHSS has not as yet been studied. The present study aimed to investigate the potential protective effects of CoQ10 on ovarian histological and molecular alterations in an experimental model of OHSS in rats.

**Materials and Methods:**

Thirty female (2 months old) Wistar rats were randomly divided into 6 equal groups: control, OHSS, OHSS+CoQ10 (OHSS+ 200 mg/kg CoQ10 for 10 days), OHSS+ cabergoline (CAB) (OHSS+ 100 µg/kg CAB for 6 days), and CoQ10 and CAB (rats receiving similar doses to treatment groups.( In the end, the effects of treatments were assessed by measuring expressions of vascular endothelial growth factor (VEGF) and cyclooxygenase-2 (COX-2) in the ovary via western blotting, ovarian histomorphological alterations assessments, and serum estradiol and progesterone levels via ELISA.

**Results:**

There were histological alterations in OHSS groups, including the elevation of diameter and numbers of the corpus luteum and atretic follicles, and decreasing follicular reserve count, hyperemia, and hemorrhage at ovarian stroma. Treatment of OHSS groups with CAB and CoQ10 could decrease histological changes, serum estrogen and progesterone, and overexpression of VEGF and COX-2 proteins.

**Conclusion:**

Our results showed that ovarian histological and molecular alterations observed in experimental OHSS can be ameliorated by administration of CoQ10, indicating that CoQ10 can be used as new supportive care for OHSS patients.

## Introduction

Female infertility is the inability to get pregnant and have a successful pregnancy (1). Ovarian Hyperstimulation Syndrome (OHSS) is a reproductive disorder that occurs in women who use injectable hormonal drugs to stimulate the ovaries to ovulate (1-3). OHSS is an exaggerated response of the ovaries to gonadotrophins. Ovarian stimulation is used to enhance the outcome of assisted reproduction treatments. Retrieving a high number of oocytes and obtaining a maximal number of embryos permit the choice of the best embryo(s) for transfer. With the development of assisted reproductive techniques and the use of ovulation-stimulating drugs, the prevalence of OHSS and its complications such as abdominal pain, ascites, fluid accumulation outside the arteries, and increased vascular permeability have been raised (4). The pathophysiology of OHSS is not well understood, but increased capillary permeability with the resulting loss of fluid into the third space is the main pathophysiological feature. Previous findings have shown that increased secretion of vascular endothelial growth factor (VEGF) in luteinized granulosa cells of the ovary has a pivotal role in the incidence and progression of OHSS symptoms in susceptible patients (5-7). Administration of hCG raises VEGF, and this results in increased production of vasoactive substances such as COX-2 and vascular permeability (7-9). Currently, there is no safe therapy available to prevent OHSS and its treatment is the management of its symptoms and complications. Compounds that block the release or action of vasoactive angiogenic substances that increase vascular permeability would appear suitable for preventing OHSS. Cabergoline (CAB) is a dopamine receptor-2 agonist that mimics the effects of dopamine by binding to dopamine receptors (10, 11). It also can effectively inhibit VEGF-VEGF2 receptor phosphorylation and VEGF secretion, which is associated with vascular permeability, and finally prevent OHSS propagation (12). Although CAB is known for its activity in preventing OHSS, each chemical drug has side effects including hypertension, hypothyroidism, diarrhea, bleeding events, and hemorrhage, and being teratogens, precludes their use in young and otherwise healthy women (13). Coenzyme Q10 (CoQ10) is a water-soluble biomolecule found naturally in mammalian cells and plays an essential role in protecting cells from free radicals and any external factors that cause oxidative damage (14, 15). CoQ10 plays a vital role in producing ATP as an electron carrier in the respiratory chain (16, 17). The reducing effect of CoQ10 on the expression of COX-2 and VEGF has also been reported in a model of hypoxia-induced retinopathy in rats (18) and polycystic ovary syndrome in women (15). Considering the beneficial effects of CoQ10 on ovarian function and its effects on the expression of VEGF and COX-2 in previous studies, it can be hypothesized that CoQ10 similar to conventional drugs such as CAB may be effective in reducing OHSS complications. Therefore, this study aimed to evaluate the protective effects of CoQ10 on histological and molecular alterations in the ovary of rats with experimental OHSS. 

## Materials and Methods

***Experimental animals and ethics***

Thirty adult Wistar female rats (2 months of age; 150 g), which had not yet mated, were obtained from the animal house of the faculty of veterinary medicine of Shahid Chamran University of Ahvaz. All animals were acclimatized in a fully controlled standard animal facility (12:12 hr light: dark cycle at 20 ± 11 °C, 40–50% humidity) and had free access to water and food (Pars, Tehran, Iran). The ethics committee of the university approved all experimental protocols for research in animals (EE/99.3.02.11772/scu.ac.ir). Working with animals was also carried out based on the Guideline for the Care and Use of Laboratory Animals (NIH publication no. N01-OD-4-213). 

***Experimental design***

The animals were weighed and randomly divided into six groups (n=5): 

Control group: rats were given normal saline; 0.5 ml/kg by subcutaneous injection, for 5 days and 1 ml/kg of normal saline by oral gavage for the next 5 days.

OHSS group: rats were given a subcutaneous injection of 10 IU equine chorionic gonadotropin (eCG) (IBSA, Montagnola, Swiss) for four consecutive days, and a single injection of 30 IU human chorionic gonadotropin (hCG) (DaruPakhsh, Tehran, Iran) on day 5 (18, 19).

OHSS+CoQ10 group: OHSS rats were given 200 mg/kg/day of CoQ10 (Goldenlife Health, New South Wales, Australia) by oral gavage from two days before OHSS induction to 10 days after OHSS stimulation (20). 

OHSS+CAB group: OHSS rats were given 100 µg/kg/day of CAB (Shahredaru, Tehran, Iran) dissolved in 5% glucosamine by oral gavage for six days (8).

CoQ10 group: rats were given 200 mg/kg/day CoQ10 by oral gavage for ten days (20).

CAB group: rats were given 100 µg/kg/day of CAB dissolved in 5% glucosamine by oral gavage for six days (21).

**Figure 1:**
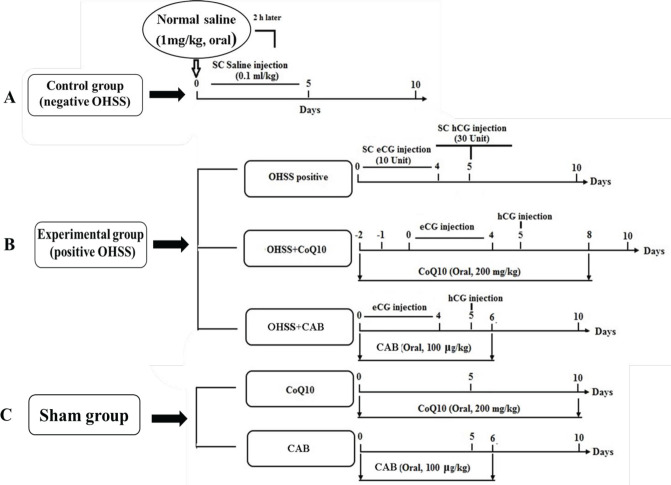
Schematic diagram of the effect of Coenzyme Q10 on a model of experimental hyperstimulation syndrome

A schematic diagram of the designed protocol is shown in Figure 1. 

OHSS induction was confirmed using the elevation of the delta value of bodyweight (difference between final body weight and initial body weight) and the elevation of estradiol (E_2_) and progesterone (P_4_) hormones as described previously (3, 22).

***Serum and tissue sample collection***

At the end of the experiment, the animals, after weighing, were euthanized using a combination of ketamine + xylazine (100 mg/kg + 10 mg/kg). The blood samples were obtained from cardiac puncture, and after clot formation, samples were centrifuged at 4000 × *g* for 5 min, and their serum was separated and stored at -20 °C until the analysis (23). The left and right ovaries were immediately removed by making an incision in the posterior abdominal area of the ventricular site. One of the ovarian samples from each rat was snap-frozen in liquid nitrogen and kept at -70 ºC until western blot analysis. Another ovary was fixed in a 10% formalin buffer (Merck, German) solution for histological examinations.

***Histomorphometric analyses***

The fixed tissue samples were prepared using standard tissue sectioning, and longitudinal sections of 5–6 μm thickness were prepared using a rotatory microtome (RM2245-LEICA, German) (24). Microscopic slides were cut at a distance of 50 microns to prevent repeated follicle counting in the ovarian tissue, and then the slides were stained with hematoxylin and eosin (Merck, Germany) dyes (25). Histological studies were performed on the prepared slides using a light microscope (Olympus BH-2, Tokyo, Japan) equipped with Dino-Eye digital lens (Dino-lite, Taiwan) and Dino Capture software (26). For grading histomorphometric alterations, five rats from each group and at least 10 microscopic fields of view from each rat were studied. The follicular reserve count (FRC), the diameter of the corpus luteum (µm), the number of corpus luteum (CL), the number of atretic follicles in the cortex, and changes in medullary tissue, including increase or decrease in vascular reserve were studied for histological analysis. Histomorphometric changes in ovaries were determined using a modified version of semi-quantified scores that was described previously by Guven *et al*. (27). The histomorphometric alteration scores were assigned as 0 = unchanged, 1 = lowest, 2 = moderate, and 3 = extensive changes based on the presence of ovarian hyperemia, hemorrhage, infiltration of mononuclear cells, interstitial tissue edema in the ovarian stroma, and changes in the nucleus of corpus luteum cells, respectively.

***Estradiol and progesterone assay***

The concentration of E_2_ and P_4_ were measured at the end of the experiment with a commercial ELISA kit according to the manufacturer’s instructions (DiaMetra, Italy). All samples were run in one assay to avoid inter-assay variation. The limits of detection of E_2_ and P_4_ were 5 pg/ml and 0.05 ng/ml, respectively. The intra assay coefficients of variation of both hormones were less than 5%. All assays were carried out in duplicates.

***Western blot analysis***

Western blot analysis was performed to evaluate the protein expression of VEGF and COX-2 in the ovaries of experimental animals. The frozen tissues were lysed in 200 μl lysis buffer (Tris-HCl 50 mM, NaCl 150 mM, Triton X-100 0.1%, NaF 1 mM) supplied with protease inhibitor cocktails (Sigma-Aldrich, MO, USA) for 30 min on ice.

The Bradford protein assay quantified the protein concentration of cell lysate. A total of 10 µg of protein was electrophoresed on a 10% SDS-PAGE. Protein was transferred onto polyvinylidene fluoride membranes and blocked overnight in 5% BSA in Tris-buffered saline with Tween 20 at 4 °C. Membranes were incubated for two hr at room temperature with the appropriate primary antibody VEGF (Abcam, Cambridge, UK, Art No: ab46154) and COX-2 (Abcam, Cambridge, UK, Art No: ab179800) diluted 1:1000 in a blocking solution. After three 15-minute washes in Tris-buffered saline with Tween 20, membranes were incubated with an appropriate secondary antibody (Goat anti-Rabbit IgG, Abcam: ab133470 (618)) diluted 1:1000 in block solution for 2 hr. After three washes, protein reactivity was visualized using an ECL detection kit (Western C; Bio-Rad). Normalized protein loading was to beta two microglobulins; B2M immunoreactivity (Abcam, Cambridge, UK, Art No: ab214769). Optical density analysis was performed using the Image J (National Institutes of Health, Bethesda, MD, USA) software processing system. The Control group was used as the calibrator group.

***Statistical analysis***

GraphPad Prism (version 5.0.4 Graph Pad Software Inc. San Diego, California, USA) software was used for the statistical analysis of data. All experiments were done in triplicate, and data are presented as the Mean±SEM. The Shapiro–Wilk or Levene’s tests were used to determine the normality of data or equality of error variances. All parameters were statistically analyzed by one-way analysis of variance (ANOVA) and Tukey multiple-comparison *post hoc* tests. Statistically significant difference between different experimental groups was represented as follows: *P<0.05, **P<0.01, *** *P*<0.001, and **** *P*<0.0001. 

## Results

***Effect of different treatments on weight changes***

The effect of different treatments on the weight change of all experimental groups is shown in Table 1. Results showed that there was a significant difference in the delta value of body weight (final weight minus initial weight) in the OHSS groups compared with the control group (129 ± 10.0 g vs 81.4 ± 3.3 g, *P*<0.001). The delta value of body weight compared with the OHSS group was significantly reduced in CoQ10- and CAB-treated groups (105.4 ± 3.0 g and 95.5 ± 2.1 g, *P*<0.05), and there was no significant difference between the two treatment groups. Rats in the OHSS group had severe ascites compared with the control group animals, characterized by distention of the abdomen and flanks. The level of ascites in rats treated with CoQ10 both and CAB showed a reduction compared with the OHSS group.

***Effect of different treatments on ovarian anatomy***

**Figure 2:**
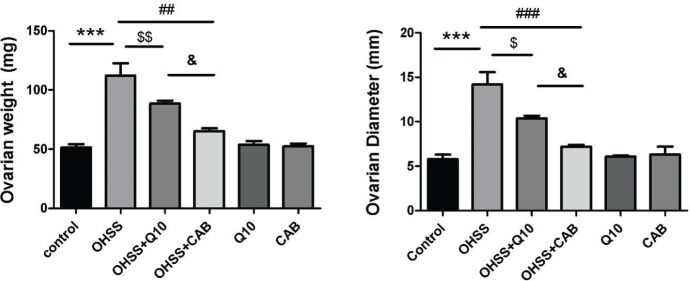
Effect of CoQ10 and CAB administration on ovarian weight and diameter (Mean ± SEM) in different experimental groups. *** indicates significant difference between OHSS and the control groups, *P*<0.001; $ indicates significant difference between OHSS+CoQ10 with the OHSS groups, *P*<0.05, double dollar signs ($$) indicate *P*<0.01; ## indicates significant difference between OHSS+CAB and the OHSS groups, *P*<0.01, triple number signs (###) indicate *P*<0.001; & indicates significant difference between OHSS+CoQ10 and the OHSS+CAB groups, *P*<0.05

CoQ10: Coenzyme Q10; CAB: Cabergoline; OHSS: Ovarian hyperstimulation syndrome

As shown in Figure 2, induction of an experimental model of OHSS caused a significant increase in ovarian weight (112.2 ± 10.3 mg vs 51.4 ± 2.8 mg, *P*<0.001) and diameter (14.2± 1.4 mm vs 5.8± 0.5 mm, *P*<0.001) compared with control group. Ovarian weight and diameter were significantly decreased in the CoQ10- and CAB-treated groups compared with the OHSS, untreated group (*P*<0.01). Our results showed that both studied parameters were significantly lower in the OHSS+CAB group compared with the CoQ10-treated OHSS group (65.2± 2.4 mg vs 88.5± 2.6 mg, *P*<0.05; 7.2 ± 0.2 mm vs 10.4± 0.3 mm, *P*<0.05, respectively).

***Effect of different treatments on ovarian histomorphology***

**Figure 3:**
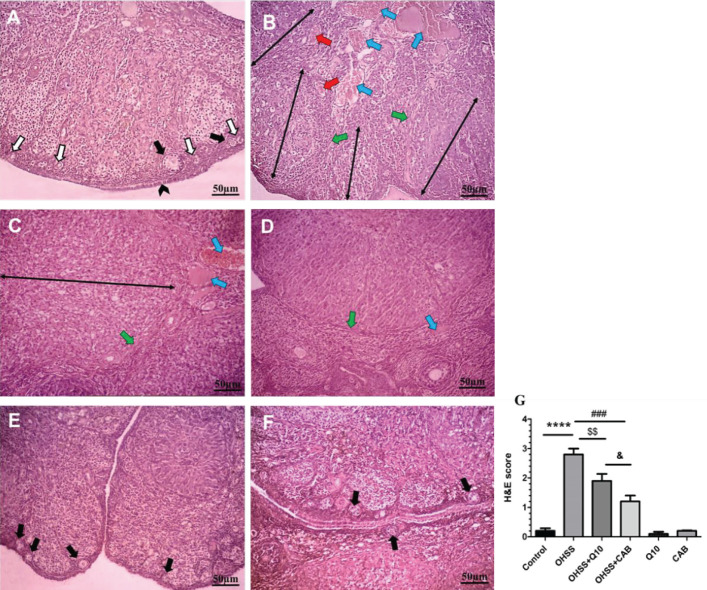
Effect of CoQ10 and CAB administration on histological changes in the OHSS rats

**Figure 4:**
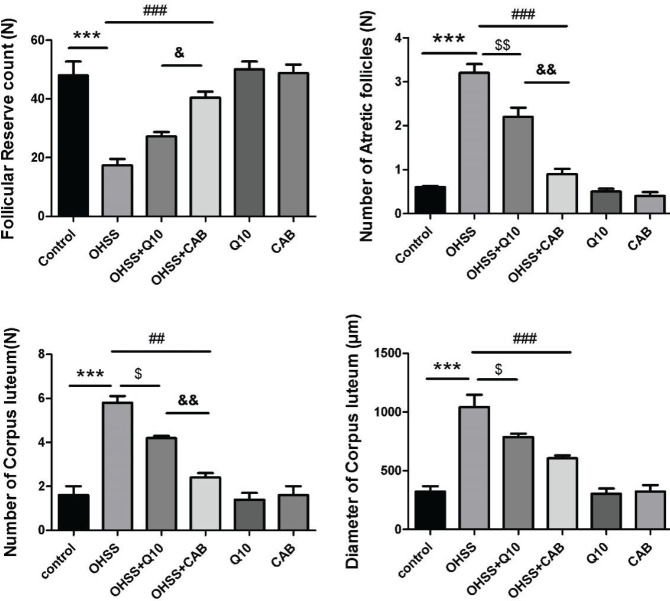
Effect of CoQ10 and CAB administration on follicular reserve count (A), the number of atretic follicles in the cortex (B), the number of corpus luteum (C), and the diameter of corpus luteum (D) of the OHSS rats. Each bar represents the mean ± SEM of 5 animals per group

The histomorphological parameters assessed in this study are shown in Figures 3 and 4. Examination of the ovarian tissue under a light microscope showed that the ovary in the control group was surrounded from the outside by a layer of simple cuboidal epithelial tissue and below that was a narrow irregular dense connective tissue. The ovarian parenchyma in the cortex consists of ovarian follicles at different stages of development located around the ovary. The primary follicle was defined as a layer of cuboidal cells around the oocyte, and the secondary follicle around the oocyte had an epithelium consisting of multilayered granulosa cells. In the ovarian medulla part, was located normal vascular tissue, connective tissue, and interstitial glandular cells (Figure 3A). Experimental induction of OHSS resulted in thinning of the tunica albuginea around the ovary compared with the control group. A thick connective tissue was pulled from the capsule into the stroma. When the ovaries of OHSS group were compared with the control group, high numbers of corpus luteum (5.8 ± 0.3 vs 1.6 ± 0.4, *P*<0.001) and atretic follicles (3.2 ± 0.2 vs 0.6 ± 0.0, *P*<0.001) were seen, but the follicular reserve count (17.4± 2.1 vs 48± 4.2, *P*<0.001) was significantly decreased. Also, the corpus luteum in group OHSS had a significantly higher diameter than those in the control group (1043.6 ± 164.9 µm vs 324.6 ± 43.3 µm, *P*<0.001). The number and diameter of corpus luteum significantly decreased in the CoQ10- and CAB-treated group compared with OHSS. The CAB-treated group had a reducing effect on the number of corpus luteum compared with the CoQ10-treated group (*P*<0.01); however, there was no significant difference in the diameter of corpus luteum between the two treatment groups. The number of atretic follicles in the cortex of the ovary in treatment groups, CoQ10- and CAB-treated, significantly decreased compared with the OHSS group. The number of atretic follicles was significantly lower in the CAB-treated group compared with the CoQ10-treated group (*P*<0.01). The follicular reserve count index in CoQ10- and CAB-treated groups were elevated compared with the OHSS group; although there was no significant difference between the two treatment groups (*P*<0.05) (Figure 4). 

Degeneration of the nucleus of lutein cells, vacuole formation in the cytoplasm, extensive angiogenesis, and increased diameter was also observed in the corpus luteum. In the medulla part of the ovarian OHSS group, the number and diameter of blood vessels were increased, and moderate to extensive hyperemia and hemorrhage were seen (Figure 3G). 

A remarkable decrease in the number of corpus luteum, atretic follicles, and the follicular reserve count was observed in both CoQ10- and CAB-treated groups (Figures 3C, D). The ovaries in the group of healthy rats receiving CoQ10 and CAB showed a tissue structure similar to the control group (Figures 3E, F).

***Effect of different treatments on serum concentrations of E***_2_ ***and P***_4_

**Figure 5:**
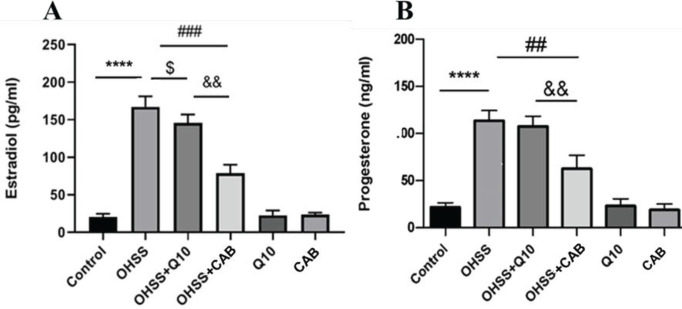
CoQ10 and CAB administration on serum concentrations of E2 (A) and P4 (B) in different experimental groups. Each bar represents the Mean ± SEM of 5 animals per group. ****: indicates significant difference between OHSS and the control groups, *P*<0.0001; $: indicates significant difference between OHSS+CoQ10 and the OHSS groups, *P*<0.05; ##: indicates significant difference between OHSS+CAB and the OHSS groups, *P*<0.01, triple number signs (###) indicate *P*<0.001; &&: indicates significant difference between OHSS+CoQ10 and the OHSS+CAB groups, *P*<0.01

CoQ10: Coenzyme Q10; CAB: Cabergoline; OHSS: Ovarian hyperstimulation syndrome

The serum concentrations of E_2_ and P_4_ in all groups are shown in Figure 5. Statistical analysis showed that the plasma E_2_ concentration was significantly increased in OHSS rats compared with the control group (167.2±13.9 pg/ml vs 20.6±4.1 pg/ml, *P*<0.0001). Administration of CAB or CoQ10 in OHSS animals caused a significant attenuation in serum E_2_ concentration. Statistical analysis showed that CAB had a more significant decreasing effect on the reduction of E_2_ compared with the CoQ10 treated group (*P*<0.01).

It was found that the level of serum P_4_ in the OHSS group was significantly increased compared with the control group (113.7±10.9 ng/ml vs 21. 9±4.6 ng/ml, *P*<0.0001). Administration of CoQ10 and CAB in OHSS groups caused a significant reduction in serum P_4_ concentration (*P*<0.01). Plasma P_4_ levels showed no significant difference between CoQ10 and CAB treated groups. The levels of E_2_ and P_4_ in the healthy groups receiving CoQ10 and CAB showed no significant difference from those in the control group (*P*>0.05).

***Effect of different treatments on ovarian expression of VEGF and COX-2***

**Figure 6:**
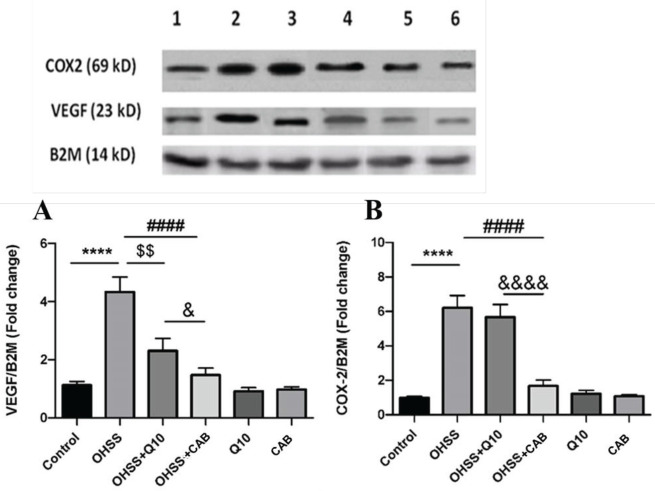
Effect of CoQ10 and CAB administration on protein expression of VEGF and COX-2 in ovarian tissue of all experimental groups. B2M was used as calibrator protein. Each bar represents the Mean ± SEM of 5 animals per group. ****: significant difference between OHSS and the control groups, *P*<0.0001; $$: Significant difference between OHSS+CoQ10 and the OHSS groups, *P*<0.01; ####: significant difference between OHSS+CAB and the OHSS groups, *P*<0.0001; &: Significant difference between the OHSS+CoQ10 and the OHSS+CAB groups, *P*<0.05, quadruple ampersand symbols (&&&&) indicate *P*<0.0001

CoQ10: Coenzyme Q10; CAB: Cabergoline; OHSS: Ovarian hyperstimulation syndrome

The expression of COX-2 and VEGF in all experimental groups is shown in Figure 6. Statistical analysis showed that VEGF expression in the OHSS group was significantly increased compared with the control group (4.3 ± 0.51 vs 1.12 ± 0.13, *P*<0.001). Administration of CoQ10 or CAB in OHSS groups caused a significant reduction in ovarian VEGF expression compared with OHSS, untreated groups. CAB treated group had a lower reducing effect on the expression of ovarian VEGF of OHSS groups compared with CoQ10 (*P*<0.05). 

Western blot analysis showed that the expression of COX-2 in the OHSS group was higher than that in the control group (6.2±0.7 vs 0.99±0.08, *P*<0.001). Administration of CAB in the OHSS group had a significant reducing effect on the ovarian COX-2 expression in relation to OHSS, untreated group (*P*<0.001). CoQ10 had no obvious reducing effect on elevated levels of COX-2 protein in the ovary of the OHSS group. The expression levels of VEGF and COX-2 showed no significant difference in control, healthy animals.

**Table 1:** The effect of different treatments on the weight changes of all experimental groups (Mean±SEM, n= 5)

CAB	CoQ10	OHSS+CAB	OHSS+CoQ10	OHSS	Control	Parameters	
**4.35** ^a^ **±** **83.8**	3.6^a^±80.6	2.10^b^±95.5	3.04^b^±105.45	10.04^c^±129	3.32^a^±81.4		**DBW (g)**

Different letters denote differences among groups at *P*<0.05; DBW: delta values of body weight (g)

“A–F” illustrate the revealing photographs of ovarian histology in different experimental groups (H&E stain, 100×magnification, scale bar = 50.0 μm). The control group (A): normal ovary displayed follicles at various stages and the presence of corpora lutea; OHSS (B): demonstrates higher numbers of the atretic follicle, corpora lutea, hyperemia in the corpus luteum, and the ovarian stroma; OHSS rats treated by CoQ10 and CAB) C and D) exhibited lower numbers of corpora lutea, atretic follicle, and tissue injuries; Healthy rats were given CoQ10 and CAB (E and F) which showed normal ovarian tissue. Arrowhead represents the superficial lining tissue of the ovary, the double-sided arrow represents a corpus luteum, the white arrow represents the primordial follicle, the black arrow represents the primary follicle, a green arrow indicates the thickening of the connective tissue wall around the follicle, the red arrow indicates hyperemia in the corpus luteum, the blue arrows indicate hyperemia in the ovarian stroma.

"G" represents the quantitative data of the histomorphometric changes. Data are presented as mean ± SEM from ten sections per rat and five animals per group. ****P<0.0001 between the OHSS and the control groups, $$ *P*<0.01 between OHSS+CoQ10 with the OHSS groups, ### *P*<0.001 between OHSS+CAB and the OHSS groups, & *P*<0.05 between OHSS+CoQ10 with OHSS+CAB groups

CoQ10: Coenzyme Q10; CAB: Cabergoline; OHSS: Ovarian hyperstimulation syndrome

***: indicates significant difference between OHSS and the control groups, *P*<0.001; $: indicates significant difference between OHSS+CoQ10 with the OHSS groups, *P*<0.05, double dollar signs ($$) indicate *P*<0.01; ##: indicates significant difference between OHSS+CAB and the OHSS groups, *P*<0.01; triple number signs (###) indicate *P*<0.001; &: indicates significant difference between OHSS+CoQ10 with OHSS+CAB groups, *P*<0.05, double ampersand symbols (&&) indicate *P*<0.01

CoQ10: Coenzyme Q10; CAB: Cabergoline; OHSS: Ovarian hyperstimulation syndrome

## Discussion

Ovarian hyperstimulation syndrome is caused by the use of assisted reproductive techniques in an attempt to increase the groups of mature and mature follicles and normal ovulation (22, 26). This syndrome is associated with increased vascular permeability and secretion of VEGF from gonadotropins stimulated ovaries during the use of one adjuvant therapy. Over secretion of VEGF can induce COX-2 expression and angiogenesis in the ovary of patients under gonadotropin therapy (6, 8). CoQ10 is a lipid-soluble electron transporter that acts as an electron carrier in the mitochondrial respiratory chain, and its function is essential for normal cellular respiration (14). Recent findings demonstrated that CoQ10 has beneficial effects on ovarian functions in normal and pathological conditions but limited data are available about the efficiency of this compound against the detrimental effects of OHSS on the ovary. Therefore, here we present novel data showing the beneficial effects of CoQ10 on the histological and molecular alterations of the ovary in an experimental model of OHSS in rats. The results of the present study showed that induction of OHSS caused an increase in rats’ weight compared with the control group. Administration of CoQ10 in OHSS groups could reduce the weight gain compared with untreated OHSS rats. This result was in accordance with previous reports that have shown the weight-lowering effect of CAB in OHSS syndrome (11, 21, 28, 29). Results of the current study indicated that the ovaries in our model have been significantly enlarged and heavier than the control group. Following CoQ10 or CAB treatment, a decrease was observed in the weight and diameter of the ovaries, but there was no significant difference between the two treatment groups (OHSS+CoQ10, OHSS+CAB). In line with our results, Hortu *et al*. indicated that following the development of OHSS syndrome, the ovaries’ weight and diameter increased, and treatment with CAB and oxytocin can reduce these effects (9). In our study, the number and diameter of corpus luteum and the number of atretic follicles of ovarian tissue were higher in the OHSS group compared with those in the control group. These changes were consistent with the findings of previous research (3, 10, 29, 30). Our findings indicated that treatment of OHSS groups by CoQ10 and CAB could reduce the increased number of corpus luteum, atretic follicles, and tissue changes. The results of the current study, for the first time, indicated that OHSS syndrome can be treated in the CoQ10-treated group, as the clinical symptoms and progression of the syndrome were attenuated in this group. As previously described, CoQ10 is an essential electron transport chain component and plays a pivotal role in cellular energy yield. Several cellular properties of CoQ10, including antioxidant (20), anti-thrombotic (31), anti-inflammatory properties (32), and anti-angiogenic effects (33) were used to treat many diseases, potentially. CoQ10 is naturally occurring which is synthesized by mammals and plants. In humans and most mammals, the predominant form of coenzyme Q is CoQ10 having 10 isoprene units in the side chain (15). Our findings agree with previous reports that showed VEGF and COX-2 protein expression were elevated in the ovaries of the OHSS rat model compared with the control group (5, 18, 34- 36). The expression of these proteins was reduced in CoQ10 and CAB-treated groups compared with the OHSS group. VEGF is the main angiogenic factor and essential mediator for OHSS, promoting endothelial cell proliferation, migration, and vascular permeability. VEGF stimulates angiogenesis through binding to VEGF receptor 2 (VEGFR2 or kinase insert domain receptor, KDR), in endothelial, granulosa, and theca cells (6, 9; 12, 36-38). Angiogenesis plays an essential role in the proliferation and metastasis of cancers, diseases’ onset, and progression (20). Administration of hCG significantly raises VEGF and VEGFR2, and this results in increased vascular permeability (39). In the current study, we established the experimental OHSS model by using an injection of hCG (5, 20). In our results, the plasma levels of E_2_ and P_4_ in the OHSS group were higher than in the control group. These findings, were in accordance with previous reports, confirming the creation of experimental OHSS in studied animals (1, 8, 35). CoQ10 is an ethical drug that has low toxicity in mammals and no genotoxic potential. Here, we found that taking CoQ10 as a nutraceutical for 10 days in subjects with OHSS can down-regulate complications of the syndrome compared with the CAB chemical drug. Presently, CAB is the most appropriate first-line treatment for this syndrome; nonetheless, side effects occur during treatment (5, 8, 9, 22). The most common adverse event in patients treated with CAB is nausea associated with/without vomiting, followed by headache, dizziness or vertigo, diarrhea, drowsiness, somnolence, paresthesia, and dyspnea (13, 40, 41). In line with this discussion, Schade 2007 indicated that the use of CAB in patients with Parkinson’s disease has a negative effect on cardiac valvulopathy (40). According to the review of articles published in prolactinoma, patients treated with Dopamine Receptor Agonist Drugs (DAs) like CAB, have many different side effects such as nausea, vomiting, orthostatic hypotension, headache, psychological, gastrointestinal, and physical symptoms (41, 42). Therefore in this way, more attention should be paid to side effects. Eventually, we found that induction of OHSS resulted in an increase in angiogenesis in the ovarian medulla and particularly in the lutein cells of the corpus luteum. According to our histological, biochemical, and molecular results, one can conclude that CoQ10 shows these protective effects through anti-angiogenesis properties. This study appears to suggest that CoQ10 can effectively prevent the development of OHSS in high-risk women; however, more cases and longer follow-ups are required to confirm and assess the safety of this treatment.

## Conclusion

The results of our study, for the first time, indicated that CoQ10, a natural substance vs chemical drugs, may attenuate the OHSS symptoms by inhibition of expression of VEGF and COX-2, as well as by attenuation of histological alterations, and reduction of serum E_2 _and P_4_ concentrations. This study helps to know the ability to use CoQ10 in reducing the symptoms of patients with OHSS and can open a new gate for improving these patients.

## Authors’ Contributions

All authors contributed to the study’s conception and design. ZB did supervised the study, was funding acquisitor, wrote the original draft, and has full access to all the data in the study; MRT participated in study design, data collection, and conducted biochemistry and molecular experiments; ZGH helped conduct the study and data analysis and interpretation; ZD contributed to experimental work. All authors read and approved the final version of the article.

## Funding

This work was funded by a grant from Shahid Chamran University of Ahvaz Research Council (GN: SCU.VB1400.103). 

## Ethical Statement

All protocols of the present study were approved by the research ethics committee of Shahid Chamran University of Ahvaz (EE/99.3.02.11772/scu.ac.ir).

## Conflicts of Interest

The authors declare that they do not have any conflicts of interest.
